# Machine learning-based classifiers to predict metastasis in colorectal cancer patients

**DOI:** 10.3389/frai.2024.1285037

**Published:** 2024-01-24

**Authors:** Raheleh Talebi, Carlos A. Celis-Morales, Abolfazl Akbari, Atefeh Talebi, Nasrin Borumandnia, Mohamad Amin Pourhoseingholi

**Affiliations:** ^1^Department of Pure Mathematics, Lecturer of Mathematics at Architecture and Computer Engineering Department, University of Applied Sciences and Technology (Unit 10), Tehran, Iran; ^2^School of Cardiovascular and Metabolic Health, University of Glasgow, Glasgow, United Kingdom; ^3^Human Performance Laboratory, Education, Physical Activity and Health Research Unit, Universidad Católica del Maule, Talca, Chile; ^4^Colorectal Research Center, Iran University of Medical Sciences, Tehran, Iran; ^5^British Heart Foundation Cardiovascular Research Centre, University of Glasgow, Glasgow, United Kingdom; ^6^Urology and Nephrology Research Center, Shahid Beheshti University of Medical Sciences, Tehran, Iran; ^7^Gastroenterology and Liver Diseases Research Center, Research Institute for Gastroenterology and Liver Diseases, Shahid Beheshti University of Medical Sciences, Tehran, Iran

**Keywords:** colorectal cancer, machine learning, metastasis, model performance and validation, balance data

## Abstract

**Background:**

The increasing prevalence of colorectal cancer (CRC) in Iran over the past three decades has made it a key public health burden. This study aimed to predict metastasis in CRC patients using machine learning (ML) approaches in terms of demographic and clinical factors.

**Methods:**

This study focuses on 1,127 CRC patients who underwent appropriate treatments at Taleghani Hospital, a tertiary care facility. The patients were divided into training and test datasets in an 80:20 ratio. Various ML methods, including Naive Bayes (NB), random rorest (RF), support vector machine (SVM), neural network (NN), decision tree (DT), and logistic regression (LR), were used for predicting metastasis in CRC patients. Model performance was evaluated using 5-fold cross-validation, reporting sensitivity, specificity, the area under the curve (AUC), and other indexes.

**Results:**

Among the 1,127 patients, 183 (16%) had experienced metastasis. In the predictionof metastasis, both the NN and RF algorithms had the highest AUC, while SVM ranked third in both the original and balanced datasets. The NN and RF algorithms achieved the highest AUC (100%), sensitivity (100% and 100%, respectively), and accuracy (99.2% and 99.3%, respectively) on the balanced dataset, followed by the SVM with an AUC of 98.8%, a sensitivity of 97.5%, and an accuracy of 97%. Moreover, lower false negative rate (FNR), false positive rate (FPR), and higher negative predictive value (NPV) can be confirmed by these two methods. The results also showed that all methods exhibited good performance in the test datasets, and the balanced dataset improved the performance of most ML methods. The most important variables for predicting metastasis were the tumor stage, the number of involved lymph nodes, and the treatment type. In a separate analysis of patients with tumor stages I–III, it was identified that tumor grade, tumor size, and tumor stage are the most important features.

**Conclusion:**

This study indicated that NN and RF were the best among ML-based approaches for predicting metastasis in CRC patients. Both the tumor stage and the number of involved lymph nodes were considered the most important features.

## Background

Colorectal cancer (CRC) has been regarded as one of the four most common types of cancer as well as the second leading cause of cancer deaths (Siegel et al., 2016). While there have been promising advancements in reducing the incidence of CRC, both morbidity and mortality rates remain high (Wieszczy et al., 2020). CRC has been steadily increasing worldwide since the 1960s, with mortality rates varying significantly according to geographical locations (Ferlay et al., 2019).

Machine learning (ML) models have become essential tools for identifying individuals at an increased risk of developing colorectal cancer and uncovering risk factors associated with the disease (Kourou et al., 2015). The ML algorithms have revealed magnificent performance in predicting survival cancers and their metastasis history. The ML-based approaches represent innovative and practical models for effectively predicting overall survival (OS) among CRC patients (Manilich et al., 2011; Wen et al., 2021). The ML methods overcome challenges in estimating coefficients and accurately modeling data. Additionally, ML models can automatically handle noise in datasets, non-linearity, complex interactions, large sample sizes, and numerous features. Overall, ML approaches have shown promise in improving treatment outcomes in cancer research (Zhou et al., 2021; Greener et al., 2022; Talebi et al., 2023). ML-based approaches have also been employed to predict metastatic relapse in breast cancer in several studies (Tapak et al., 2019; Nicol et al., 2020). Furthermore, numerous epidemiological studies have investigated specific hypotheses related to CRC risk factors (Talebi et al., 2019, 2020; Borumandnia et al., 2021). These studies encompass survival analysis techniques such as Cox proportional hazards, time-dependent Cox, cure models, and other types of survival analysis using clinical datasets. Moreover, other investigations have utilized ML models such as decision tree (DT), support vector machine (SVM), neural network (NN), and Naive Bayes (NB) methods (Talebi et al., 2023).

This historical cohort study aims to predict CRC survival using supervised machine learning methods, including NB, random forest (RF), SVM, NN, DT, and logistic regression (LR).

## Methods

Clinical data from 1,127 patients who underwent medical treatment for rectal cancer at Taleghani Hospital, a tertiary care facility, from 2013 to 2019, were scrutinized to develop prediction models using an ML classifier. Metastasis served as the dependent variable, while demographic and clinical factors were considered as independent variables. Demographic characteristics included sex, age, education level, smoking, marital status, and BMI, while clinical factors encompassed the number of involved lymph nodes, tumor grade, tumor stage, treatment type, and diabetes mellitus.

This research adheres to the principles of the Helsinki Declaration. The methods were conducted in accordance with relevant guidelines and official instructions. The ethics committee of Iran University of Medical Sciences has waived the requirement for informed consent and approved the project (IR.IUMS.REC.1400.459).

### Inclusion and exclusion criteria

The inclusion criteria for the CRC screening program, which spanned from 2013 to 2019, included various diagnostic methods such as endoscopy, imaging, stool, or blood tests, and colonoscopy reports for patients diagnosed with CRC from 2013 to 2019. Additionally, the clinical data were utilized for assessing the overall or relative survival of CRC patients who had experienced metastasis. On the contrary, the exclusion criteria included the absence of a colonoscopy report or incomplete data.

### Preliminary processing of data

**Figure 1:**
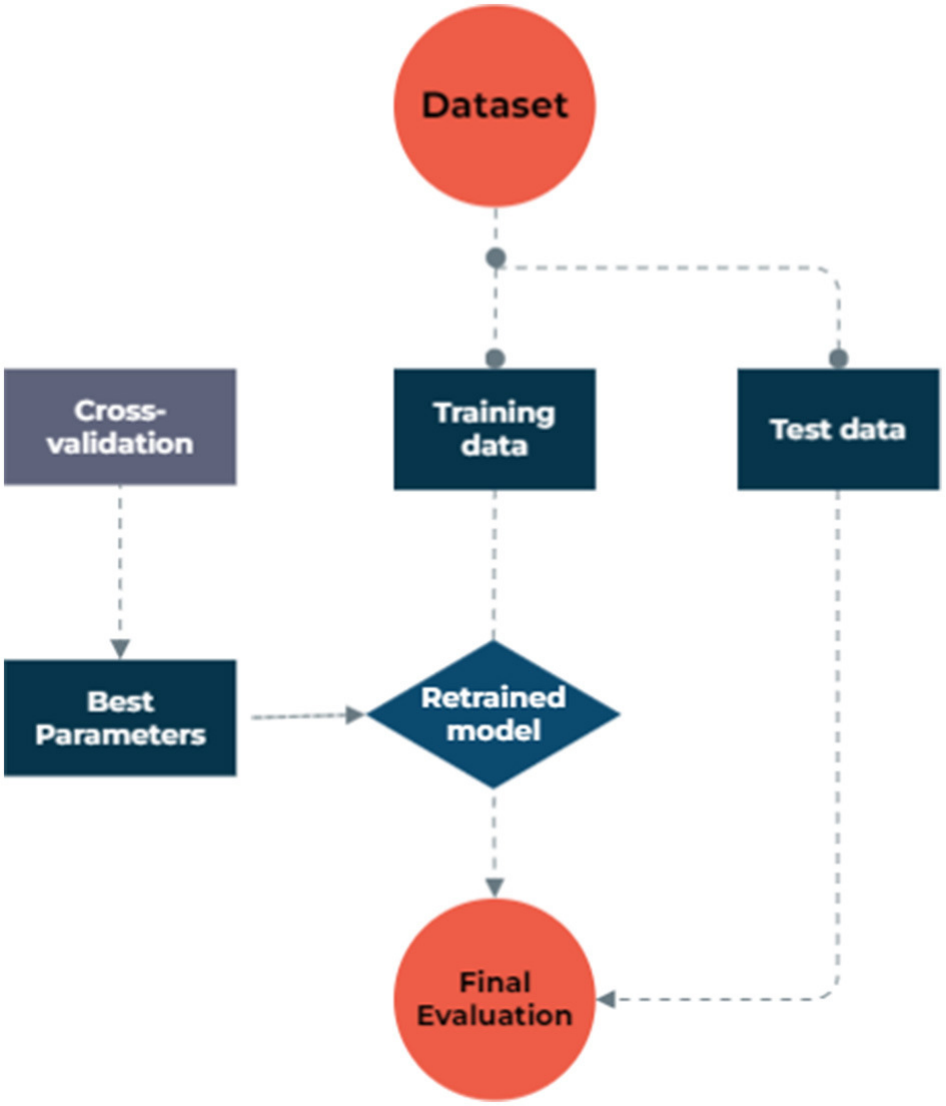
The model selection process.

To address missing data, we utilized model-based imputation techniques. The dataset used in the study consisted of 1,127 samples and 13 factors, including patients' demographic and clinical characteristics, as well as their metastasis status as an outcome binary variable, obtained from archived records. To facilitate the analysis, categorical features were transformed into discrete values using binning discretization methods. The continuous variables were normalized through one-hot encoding, centering them around the mean and scaling to a standard deviation of 1. In the NB model process, we grouped numeric values into four equally frequent categories. The dataset exhibited a significantly imbalanced distribution of metastasis rates, with a ratio of 16 metastasis to 84 non-metastasis cases, which could lead to biased models that favor the majority class and ignore the minority class, resulting in poor sensitivity and precision. To overcome this issue, the synthetic minority oversampling technique (SMOTE) was employed to balance the data by creating synthetic samples from the minority class. The SMOTE created synthetic samples from the minority class by finding the k-nearest neighbors of each sample and randomly choosing one of them to create a new sample along the line connecting them, which led to a new balanced dataset. Both the original and balanced datasets were then used to implement various ML algorithms, and their results were compared in terms of model performances. Figure 1 illustrates the step-by-step data processing and model selection process.

### Model development

ML models were developed using the five-fold cross-validation method to assess the impact of the selected variables. The dataset was divided into five randomly selected folds, with 4-folds used for model training and the remaining fold applied for testing. This process ensured that 80% of the data was used for training and 20% for testing in each iteration. Various algorithms, including NB, RF, SVM, NN, DT, and LR, were employed to create models for predicting metastasis in CRC patients. The hyperparameters were tuned using a grid search technique, which involves testing different combinations of hyperparameter values and selecting the best one based on the cross-validation performance and prior experience. The performance of the models was evaluated using the validation data, and this iterative process was repeated until satisfactory results were obtained. In the case of the NN approach, algorithm selection was done through trial and error. A multi-layer perceptron network with the rectified linear unit activation function and stochastic gradient-based optimizer for weight optimization was utilized for NN modeling. For DT, a forward pruning technique was employed to split the data based on class purity. The RF algorithm constructed a set of decision trees using bootstrap sampling from the training data. The SVM algorithm employed a radial basis function kernel in this study. The modeling process for SVM included setting the cost to 1.00, regression loss to 0.1, and numerical tolerance to 0.001. Additionally, an NB classifier based on the Bayes' theorem was fitted. This method is known for its speed and robust performance.

### Statistical analysis

The characteristics of participants were presented by reporting the mean ± SD for continuous variables and frequency with percentage for categorical ones. Missing data were imputed using model-based imputation methods. The ML-based approaches, including NB, RF, SVM, NN, DT, and LR, were used for predicting metastasis in CRC patients. The data were divided into training and test datasets in an 80:20 ratio. Then, the performance of various ML methods on both the original and balanced datasets was compared using 5-fold cross-validation and ROC curves. The area under the curve (AUC) of ROC, Precision–Recall area under the curve (PR-AUC), sensitivity, specificity, false negative rate (FNR), false positive rate (FPV), negative predictive value (NPV), F1 score, accuracy, and precision were reported for both the training and test sets in both the original and balanced datasets. The training accuracy is the overall accuracy of the model obtained by averaging the accuracies from the individual cross-validation runs. Additionally, calculating the performance analysis and obtaining the vital factors were carried out in stages I, II, and III. The ML modeling was implemented using Orange3 software version 3.36.1 and R studio version 4.2.0.

## Results

A total of 1,127 registered CRC patients who experienced metastasis in 183 (16.2%) cases were included in this historical cohort study. The mean ± SD age of patients was 53.59 ± 14.35 years, ranging from 14 to 94 years. Out of the total number of patients, 437 (38.8%) patients were women. Demographic and clinical information is provided in Table 1.

**Table 1:** Demographic and clinical attributes of patients with colorectal cancer.

**Variables**		**Total (*****n*** = **1,127)**		**Metastasis**			
				**No (*****n** =* **944)**		**Yes (*****n** =* **183)**	
Age, mean (SD)		53.59 (14.35)		53.67 (14.45)		53.20 (13.82)	
Tumor size, mean (SD)		5.34 (3.18)		5.33 (3.28)		5.40 (2.61)	
Sex	Female	437	38.8%	376	86.0%	61	14.0%
	Male	690	61.2%	568	82.3%	122	17.7%
Marital status	Married	1,050	93.2%	880	83.8%	170	16.2%
	Single	77	6.8%	64	83.1%	13	16.9%
Education	Illiterate	309	27.4%	251	81.2%	58	18.8%
	Primary school	373	33.1%	311	83.4%	62	16.6%
	High school	266	23.6%	231	86.8%	35	13.2%
	University	179	15.9%	151	84.4%	28	15.6%
BMI	<18	79	7.0%	65	82.3%	14	17.7%
	18–25	641	56.9%	521	81.3%	120	18.7%
	>25	407	36.1%	358	88.0%	49	12.0%
Smoking	No	841	74.6%	719	85.5%	122	14.5%
	Yes	286	25.4%	225	78.7%	61	21.3%
Diabetes	No	1,047	92.9%	873	83.4%	174	16.6%
	Yes	80	7.1%	71	88.8%	9	11.3%
Family history	No	719	63.8%	599	83.3%	120	16.7%
	Yes	408	36.2%	345	84.6%	63	15.4%
Tumor grade	Poorly	78	6.9%	62	79.5%	16	20.5%
	Moderately	412	36.6%	336	81.6%	76	18.4%
	Well	637	56.5%	546	85.7%	91	14.3%
Number of involved lymph	N0	531	47.1%	481	90.6%	50	9.4%
node	N1	596	52.9%	463	77.7%	133	22.3%
Tumor stage	I	84	7.5%	82	97.6%	2	2.4%
	II	427	37.9%	423	99.1%	4	0.9%
	III	412	36.6%	406	98.5%	6	1.5%
	IV	204	18.1%	33	16.2%	171	83.8%
Treatment type	Other treatments	119	10.6%	80	67.2%	39	32.8%
	Surgery	1,008	89.4%	864	85.7%	144	14.3%

Subsequently, various ML algorithms were applied to predict metastasis in CRC patients. Table 2 reveals the performance of different ML algorithms, which were evaluated using a 5-fold CV. It can be explained that the performance of all methods was acceptable in clinical research. The highest sensitivity and specificity (97.9% and 98.7%, respectively) were estimated for the NN algorithm. Both NN and RF algorithms had the highest AUC, while the SVM ranked third in both the original and balanced datasets. In addition, the evaluation of the results indicated that all methods exhibited good performance in the test datasets. The results also showed that the balanced dataset improved the performance of most ML methods, especially DT and NN, in predicting metastasis in CRC patients. As shown in Table 2, the balanced dataset increased the sensitivity and AUC of most ML methods, indicating that the models were able to better distinguish between metastasis and non-metastasis cases. The NN and RF achieved the highest AUC (100%), sensitivity (100% and 100%, respectively), and accuracy (99.2% and 99.3%, respectively) on the balanced dataset, followed by the SVM with an AUC of 98.8%, a sensitivity of 97.5%, and an accuracy of 97%. Moreover, lower FNR and FPR and higher NPV can be confirmed by these two methods. These results suggest that the NN and RF models are the most suitable ML methods for predicting metastasis in CRC patients as they can capture the complex and non-linear relationships between the features and the outcome. The SVM, LR, and NB models also showed improved performance on the balanced dataset, while they were still inferior in the NN and RF models.

**Table 2:** Performance criteria for the ML methods for predicting metastasis in colorectal cancer patients.

			**AUC**	**PR-AUC**	**Accuracy**	**F1 score**	**Precision**	**Sensitivity**	**Specificity**	**FNR**	**FPR**	**NPV**
DT	Original	Train	96.8	95.2	97.7	92.3	97.7	87.5	99.6	12.5	0.4	88.8
		Test	89.5	74.8	92.5	75.4	86.7	66.7	97.9	33.3	2.1	74.6
	Balanced	Train	96.6	95.3	96.5	96.5	96.2	96.8	96.2	3.2	3.8	96.8
		Test	94.7	94.8	94.1	94.0	96.1	92.0	96.3	8	3.7	92.3
SVM	Original	Train	98.2	91.8	96.2	88.8	84.4	93.8	96.7	6.2	3.3	94.0
		Test	95.8	86.7	96.5	90.0	87.8	92.3	97.3	7.7	2.7	92.7
	Balanced	Train	98.8	97.8	97.0	97.0	96.6	97.5	96.9	2.5	3.1	97.5
		Test	95.5	95.2	94.7	94.5	97.2	92.0	97.3	8	2.7	92.4
RF	Original	Train	99.8	98.7	97.8	93.3	90.3	96.5	98.0	3.5	2	96.6
		Test	95.9	92.3	95.1	85.7	86.8	84.6	97.3	15.4	2.7	86.3
	Balanced	Train	1.0	1.0	99.3	99.3	98.7	1.0	98.7	0	1.3	100.0
		Test	94.8	95.1	90.9	90.3	97.0	84.6	97.3	15.4	2.7	86.3
NN	Original	Train	99.9	99.5	98.6	95.6	93.4	97.9	98.7	2.1	1.3	97.9
		Test	96.3	92.5	94.2	82.2	88.2	76.9	97.9	23.1	2.1	80.9
	Balanced	Train	1.0	99.9	99.2	99.2	98.4	1.0	98.4	0	1.6	100.0
		Test	96.5	97.0	89.9	89.0	97.5	81.9	97.9	18.1	2.1	84.4
NB	Original	Train	96.6	85.3	95.9	87.9	83.2	93.1	96.4	6.9	3.6	93.3
		Test	96.9	89.4	96.0	88.6	87.5	89.7	97.3	10.3	2.7	90.4
	Balanced	Train	96.4	96.0	95.1	95.1	96.2	93.9	96.3	6.1	3.7	94.0
		Test	96.5	96.6	94.7	94.5	97.2	92.0	97.3	8	2.7	92.4
LR	Original	Train	97.8	95.2	95.8	87.6	82.7	93.1	96.3	6.9	3.7	93.3
		Test	94.6	89.8	96.5	89.7	89.7	89.7	97.9	10.3	2.1	90.5
	Balanced	Train	97.9	96.7	95.7	95.7	96.3	95.1	96.3	4.9	3.7	95.2
		Test	94.5	95.5	94.4	94.3	96.6	92.0	96.8	8	3.2	92.4

NB, Naive Bayes; RF, random rorest; SVM, support vector machine; NN, neural network; DT, decision tree; LR, logistic regression; FNR, false negative rate; NPV, negative predictive value; FPV, false positive rate.

**Figure 2:**
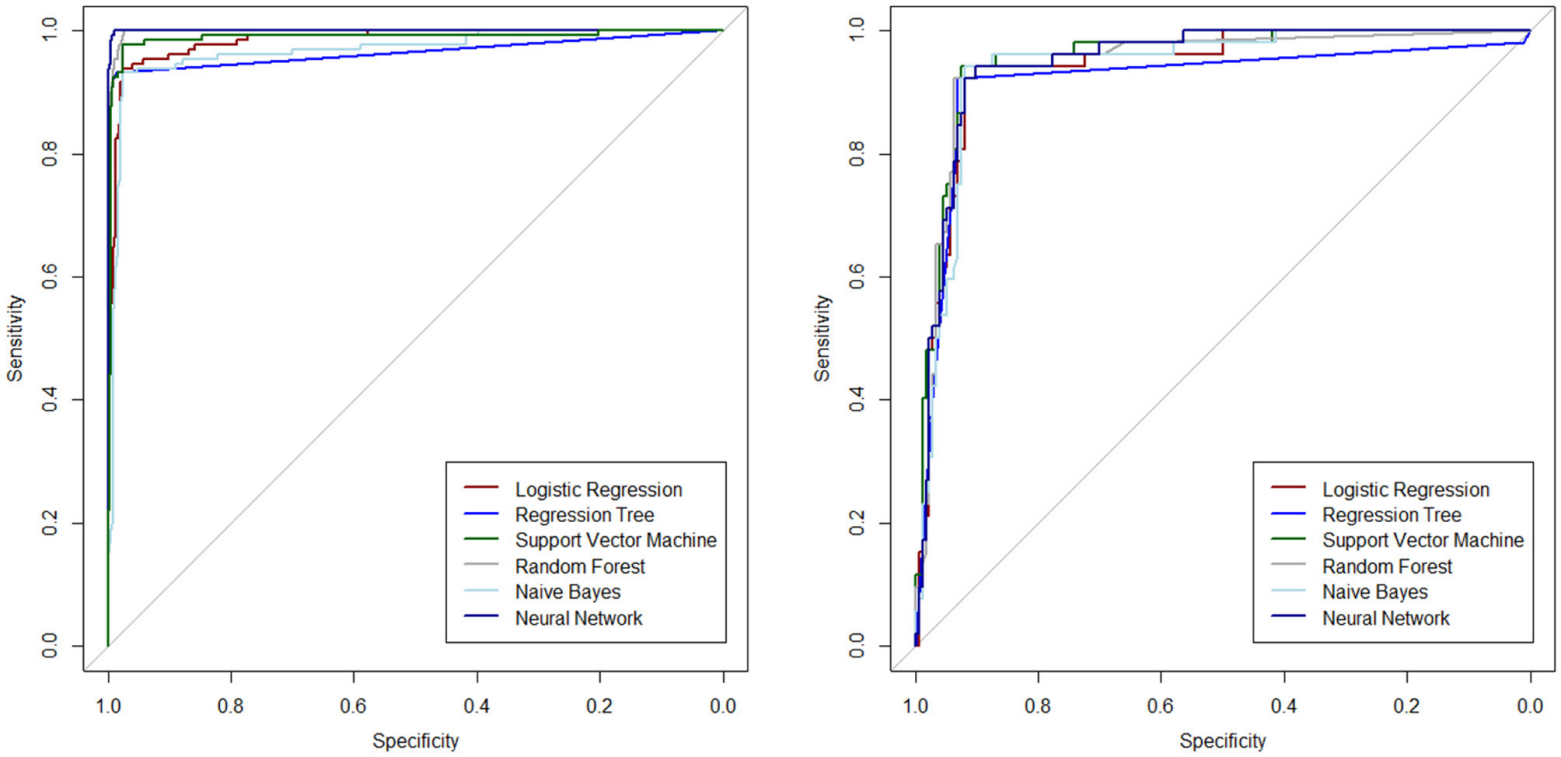
ROC curves for different ML algorithms on training **(left)** and test **(right)** datasets.

The ROC curves are plotted to determine the diagnostic ability of the ML algorithms in Figure 2, separately for the test and training datasets.

**Figure 3:**
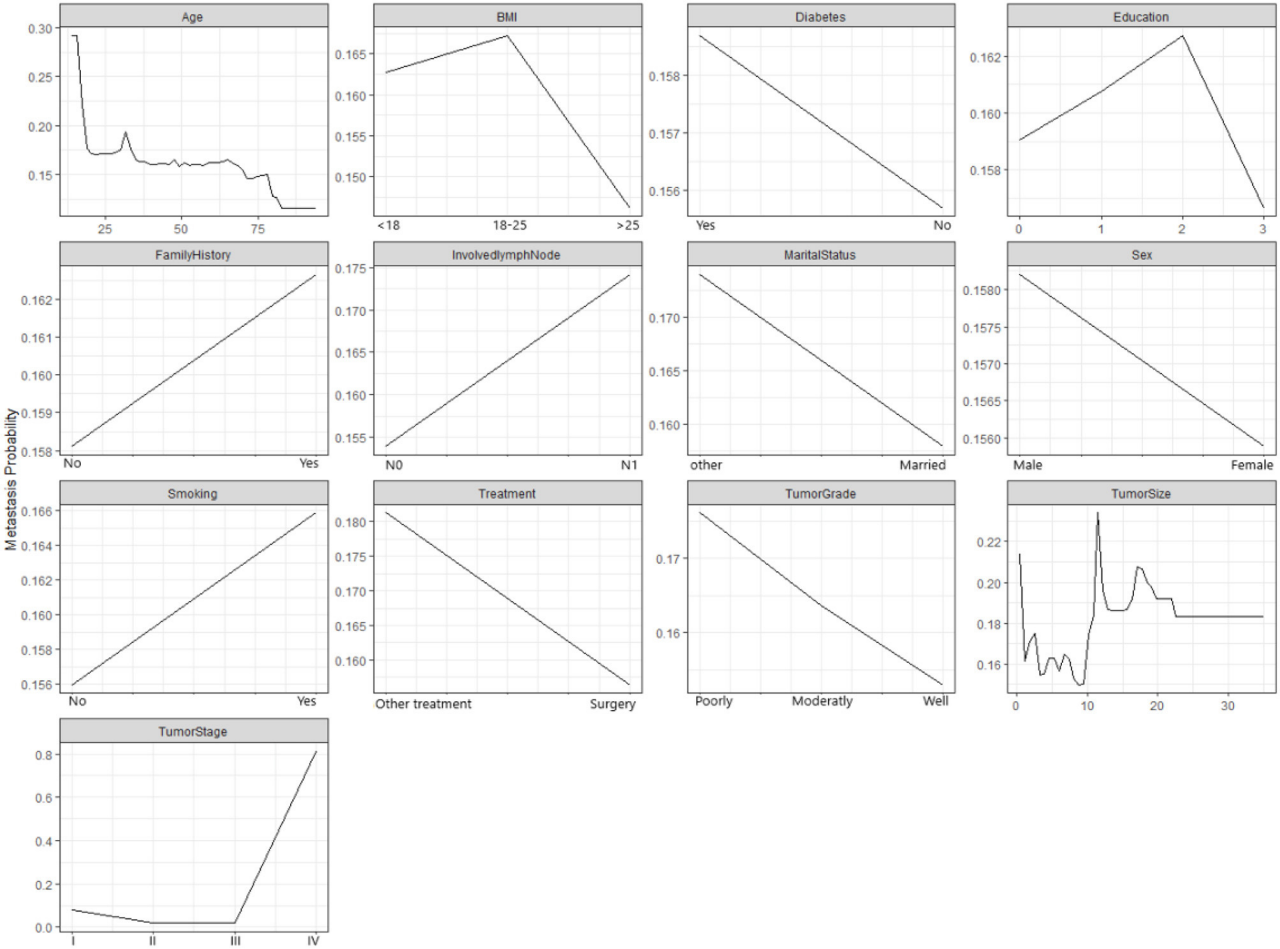
The partial relationship between the probabilty of metastasis and features.

Figure 3 demonstrates how the probability of metastasis depends on various features of the patients and their tumors. Some of the graphs show a clear relationship between the feature and the probability of metastasis, such as age, tumor size, and tumor stage. For example, the graph for the tumor stage shows that the probability of metastasis increases as the the tumor stage increases, which means that more advanced tumors are more likely to spread than less advanced tumors. Some of the graphs show a weak or unclear relationship between the feature and the probability of metastasis, such as diabetes, education, family history, and marital status. For example, the graph for diabetes shows that the probability of metastasis is slightly higher for patients who have diabetes than for patients who do not have diabetes. However, the difference is not very large.

**Figure 4:**
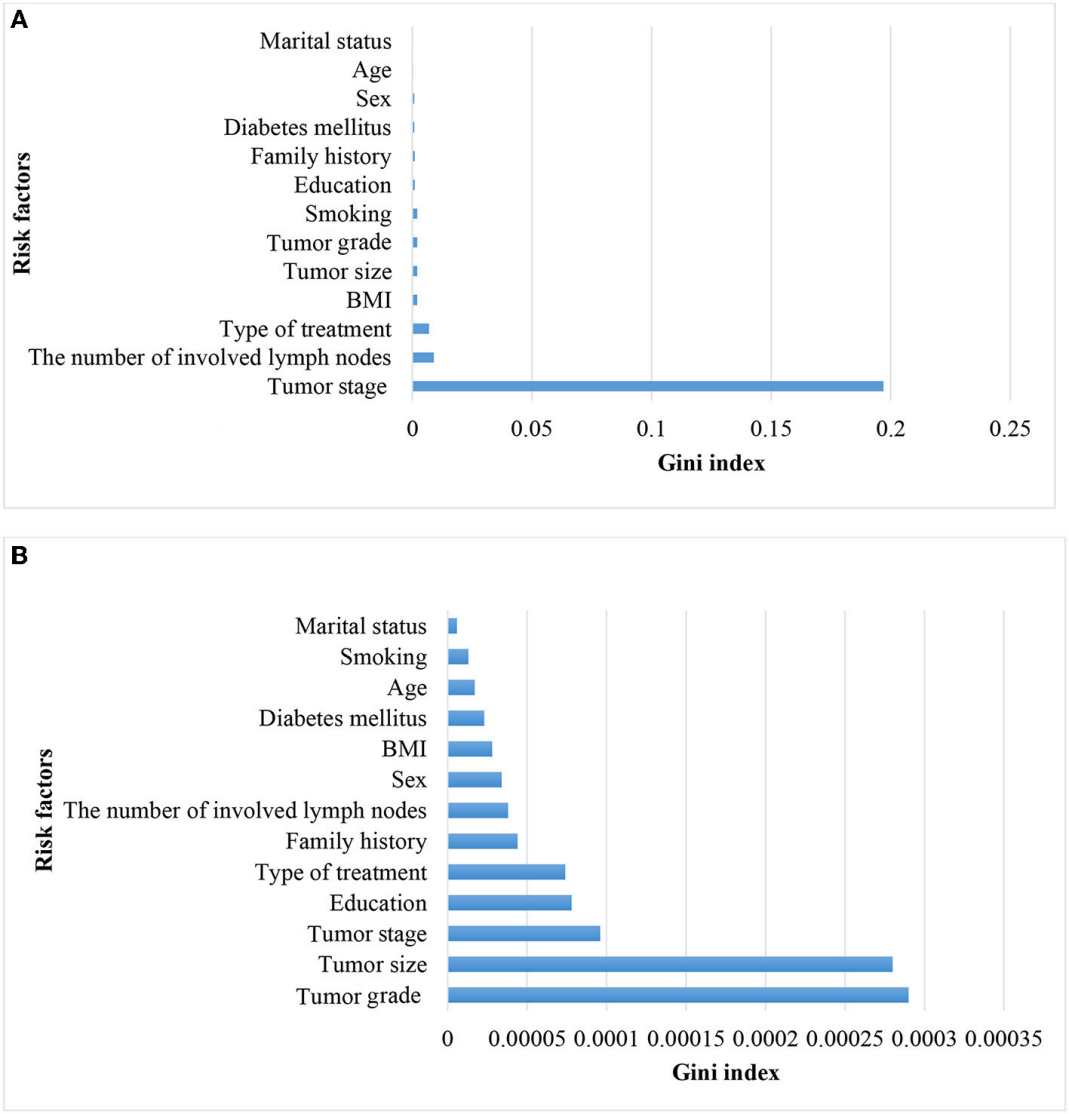
Variable importance for predicting metastasis in patients with CRC in all stages **(A)** and without CRC in stage IV **(B)**.

Considering that tumor stage IV is a strong predictor and that modeling tumor cases at earlier stages can have a significant impact on early detection, a separate analysis was conducted on the subset of patients with tumor stages I–III (935 cases, of which 12 had metastasis). The performance of the models was compared with the baseline model based on all stages. Since the performance analysis based on dropping stage IV in the original data was unreliable due to the small sample size of metastasis (12 cases, equal to 1%), only the results of balanced datasets were reported (Table 3). The two approaches, NN and RF, had good performance. The rest of the models did not perform well. Figure 4 demonstrates that the most significant variable is determined by the Gini index. Figure 4A shows that the tumor stage was the most significant factor for predicting metastasis. Subsequently, the number of involved lymph nodes, treatment type, BMI, and tumor size played key roles in predicting metastasis. Figure 4B demonstrates that tumor grade, tumor size, and tumor stage are the most important factors.

**Table 3:** Performance criteria for ML methods in predicting metastasis in the balanced dataset of patients with tumor stages I–III.

		**AUC**	**PR-AUC**	**Accuracy**	**F1 score**	**Precision**	**Sensitivity**	**Specificity**	**FNR**	**FPR**	**NPV**
DT	Train	67.2	65.3	95.4	48.6	94.4	32.7	99.9	67.3	0.1	59.7
	Test	73.8	73.1	95.9	55.6	71.4	45.5	98.9	54.5	1.1	64.5
SVM	Train	99.9	98.6	97.4	76.7	97.1	63.5	99.9	36.5	0.1	73.2
	Test	96.7	96.3	96.4	53.3	100.0	36.4	100.0	63.6	0	61.1
RF	Train	100.0	99.9	99.6	96.9	100.0	98.0	100.0	2	0	98.0
	Test	99.7	99.9	97.9	90.0	88.9	90.7	99.5	9.3	0.5	91.5
NN	Train	100.0	99.8	99.9	99.0	98.0	100.0	99.9	0	0.1	100.0
	Test	99.8	99.9	99.5	95.7	91.7	100.0	99.5	0	0.5	100.0
NB	Train	88.0	88.2	94.3	38.9	63.6	28.0	98.9	72	1.1	57.9
	Test	88.0	88.1	94.9	37.5	60.0	27.3	98.9	72.7	1.1	57.6
LR	Train	92.5	91.2	93.7	22.2	53.8	14.0	99.2	86	0.8	53.6
	Test	89.4	90.6	93.8	25.0	40.0	18.2	98.4	81.8	1.6	54.6

NB, Naive Bayes; RF, random forest; SVM, support vector machine; NN, neural network; DT, decision tree; LR, logistic regression; FNR, false negative rate; NPV, negative predictive value; FPV, false positive rate.

## Discussion

In the present study, ML models were applied to predict metastasis in CRC patients. The clinical efficacy of our models was determined through ROC curve analysis and other indices, including sensitivity, specificity, and precision. Classifier performance was assessed using the six ML-based approaches. In addition, to focus on modeling tumor cases at earlier stages, which is important for early detection, a separate analysis was conducted for the subset of patients with tumor stages I–III. However, the results of these stages may be unreliable because the number of metastasis samples was very small in these patients.

In our study, while all models exhibited acceptable performance, the NN and RF models demonstrated greater predictive efficiency than the others. A number of ML-based modeling techniques have been suggested for the CRC dataset. Alternative studies have applied DT, SVM, NN, RF, and LR (Cueto-López et al., 2019; Boyne et al., 2020; Nartowt et al., 2020; Achilonu et al., 2021). A study investigated the prediction of tumor staging in colon cancer patients using TNM staging (tumor, node, and metastasis) (Gupta et al., 2019). In this study, ML techniques, such as RF, LR, SVM, NN, k-nearest neighbor (KNN), and adaptive boosting, were applied based on grouping tumor aggression score (TAS) into two categories (>9.8 and <9.8). They concluded that, when tumor size alone was regarded as a prognostic factor, the RF model outperformed other approaches with an accuracy of 84% and 74% in the training and test sets, respectively. In our study, we performed six ML-based approaches using the CRC data, and we found that NN and RF outperformed other models. The RF model was particularly compatible with our study. Moreover, NN and RF presented the highest sensitivity; furthermore, NN and DT showed the highest values in specificity. In their study, both the tumor stage and the number of involved lymph nodes are regarded as the most significant factors. However, the tumor stage was an essential variable, which is consistent with our study.

In 2020, Boyne et al. predicted early discontinuation of adjuvant chemotherapy among individuals aged >17 years with colon cancer patients at a high stage using the LR and RF models (Boyne et al., 2020). Their results revealed that the time from surgery to chemotherapy initiation and the distance from the treatment facility seemed to be the most considerable predictor factors. They concluded that the RF algorithm may help predict early discontinuation of chemotherapy among stage III colon cancer patients. In our study, the NN and RF models were of primary and secondary importance. The primary outcome of their study was chemotherapy discontinuation, defined as a receipt of <5 months and more than 5 months, while metastasis was the dependent variable in our study. In their study, RF was considered a better model than the LR method, while all ML-based approaches exhibited ideal performances in our study.

An investigation was conducted in South Africa using LR, NB, C5.0, RF, SVM, and ANN algorithms for predictive analytics of recurrence and survival outcomes in CRC patients (Achilonu et al., 2021). The analysis considered three datasets, including simulated, recurrent, and survival data. Significant variables in all models were examined and compared using the AUC, which evaluated the discriminatory power of predictive models. This assessment was supported by a threshold (accuracy) metric. Their results demonstrated that all models had the AUC values >80%; however, the ANN model was considered a better method with an AUC of approximately 100%, which was compatible with our study. Nevertheless, an inconsistency arose in the results of the African study, where histology and CRC complications were prioritized in six methods, while in our study, the tumor stage emerged as the primary candidate.

A survey was carried out on the Indonesian population suffering from CRC in four hospitals from 2012 to 2015 (Anuraga and Fernanda, 2019). The predictor factors included the comorbidity, the tumor stage, age, treatment type, cancer location, gender, and metastasis in CRC patients. In this survey, the RF algorithm was employed in data classification, utilizing tree merging through training on sample data. Furthermore, the accuracy of these models was assessed based on the classification value using the AUC. In addition, the most essential variables for the survival of CRC patients were the metastasis history, cancer location, and gender. In our investigation, the outcome variable was metastatic history, whereas the survival of CRC patients served as the dependent variable in the Indonesian study. Moreover, in their study, both the tumor stage and age were of less importance, with the tumor stage being consistent with our survey.

There are some limitations to our study: This study was based on a single tertiary care facility in Iran, which may limit the generalizability of the results to other populations and settings. In addition, the other potential predictors of metastasis, such as the molecular markers, tumor microenvironment, and treatment response, were not considered due to the lack of data availability. The study used a binary outcome of metastasis, which may not capture the complexity of the metastatic process and its clinical implications.

## Conclusion

The results of this study indicated that the NN and RF methodscould be the best among ML-based approaches. In addition, in the RF method, the most important variables were the tumor stage, the number of involved lymph nodes, treatment type, and BMI. In a separate analysis of patients with tumor stages I–III, the performance of the NN and RF models was acceptable, with tumor grade, tumor size, and tumor stage identified as the most important factors. However, the results of modeling of I–III stages should be used with caution because the number of metastasis samples was very small.

## Data availability statement

The original contributions presented in the study are included in the article/supplementary material, further inquiries can be directed to the corresponding author.

## Ethics statement

The studies involving human data were approved by the Ethics Committee of Iran University of Medical Sciences (IR.IUMS.REC.1400.459). The Ethics Committee waived the requirement for written informed consent.

## Author contributions

RT: Investigation, Software, Writing – original draft. CC-M: Supervision, Project administration, Writing – review & editing. AA: Conceptualization, Validation, Writing – review & editing. AT: Formal analysis, Investigation, Writing – review & editing. NB: Formal analysis, Project administration, Software, Supervision, Writing – review & editing. MP: Data curation, Writing – review & editing.
